# Joining forces: a complex cardio-obstetrics case report of severe ergometrine-induced vasospasm

**DOI:** 10.1093/ehjcr/ytaf087

**Published:** 2025-02-18

**Authors:** Avinash Radhakrishna, Cathy Burke, Patrick Barry, Christian Cawley, Heather Cronin

**Affiliations:** Department of Cardiology, Cork University Hospital, Wilton Road, Cork T12 DC4A, Ireland; Department of Obstetrics and Gynaecology, Cork University Hospital, Wilton Road, Cork T12 DC4A, Ireland; Department of Geriatric Medicine, Cork University Hospital, Wilton Road, Cork T12 DC4A, Ireland; Department of Cardiology, Cork University Hospital, Wilton Road, Cork T12 DC4A, Ireland; Department of Cardiology, Cork University Hospital, Wilton Road, Cork T12 DC4A, Ireland

**Keywords:** Cardio-obstetrics, Ergometrine, Post-partum haemorrhage, Coronary artery vasospasm, Pregnancy-associated myocardial infarction, Heart failure, Case report

## Abstract

**Background:**

Ergometrine is part of the current guideline-directed management of post-partum haemorrhage (PPH). However, it is also a potent vasoconstrictor capable of causing significant coronary artery vasospasm in susceptible individuals.

**Case summary:**

A 31-year-old primigravida suffered from post-partum haemorrhagic shock and disseminated intravascular coagulation following vaginal delivery. She was urgently resuscitated with intravenous fluids and blood products. Intramuscular ergometrine was administered, and surgery was required due to retained placenta. Two days later upon extubation, she demonstrated symptoms of speech apraxia. Brain magnetic resonance imaging (MRI) revealed extensive cerebral and cerebellar infarction with subcortical sparring. Her electrocardiogram showed diffuse T-wave inversions, and a high-sensitivity troponin-I peaked at 37 000 ng/L. Echocardiography showed severe left ventricular (LV) failure, apical akinesia, and thrombi formation. One week later, she suffered a middle cerebral artery stroke causing aphasia and right-sided hemiparesis, necessitating emergency thrombectomy. Cardiovascular MRI showed moderate LV systolic impairment and focal apical infarction. Coronary angiography was unremarkable. The most likely unifying diagnosis was severe ergotamine-induced coronary vasospasm causing acute myocardial infarction in the setting of life-threatening PPH. Following three weeks of multi-disciplinary care, her speech and motor abilities improved. She was discharged on long-acting nitrates, oral anticoagulation, and heart failure therapy with close outpatient monitoring. Subsequent echocardiograms showed marked improvement in LV ejection fraction (45%–50%).

**Discussion:**

This case highlights the potential life-threatening complications of ergometrine and the importance of recognizing pregnancy-associated myocardial infarction as a significant cause of maternal morbidity and mortality. The cardio-obstetrics team plays a pivotal role in improving patient outcomes.

**Learning points**

- Although ergometrine is part of the guideline-directed management of post-partum haemorrhage, it is a potent vasoconstrictor, capable of causing profound coronary vasospasm and acute myocardial infarction.
- This case report highlights the importance to recognize pregnancy-associated myocardial infarction (PAMI) as a significant cause of maternal morbidity and mortality.
- The early involvement of the multi-disciplinary cardio-obstetrics team is crucial to establish prompt treatment strategies and manage potential complications associated with PAMI. Plans for future pregnancies should be individualized through a shared decision-making process prior to conception.

## Introduction

Ergometrine is a potent ergot alkaloid and is widely used for its efficacy in reducing post-partum blood loss. It works by stimulating the alpha-adrenergic, serotonin, and dopaminergic receptors leading to vasoconstriction and uterine muscle contraction.^1^ However, its vasoconstrictive effects may extend beyond the uterus, posing risks to coronary arteries in susceptible individuals. This case report delves into the complexities surrounding the use of ergometrine in managing post-partum haemorrhage (PPH), juxtaposed against its potential to precipitate acute myocardial infarction (AMI) through severe vasospasm.^2^

## Summary figure

**Figure:**
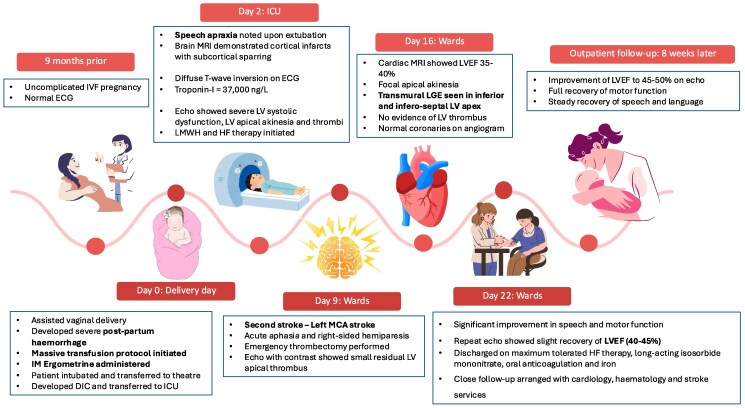


## Case presentation

A 31-year-old primigravida with an uncomplicated pregnancy presented in spontaneous labour at full-term. She has a history of recovered eating disorder and secondary amenorrhoea because of low body weight, necessitating conception through *in vitro* fertilization. Her family history revealed no predisposition to thrombotic events.

Following the delivery of a healthy daughter through assisted forceps vaginal delivery, she immediately developed haemorrhagic shock due to retained placenta. This required a massive transfusion protocol comprising 5 L of intravenous fluids, six units of packed red cell concentrates, two units of fresh frozen plasma, one pool of platelets, and 2 g of tranexamic acid. Additionally, she was given intramuscular ergometrine and intrauterine carboprost. As she became more haemodynamically unstable with an arterial lactate of 8 mmol/L (0.4–1.3 mmol/L) and haemoglobin of 7.3 g/dL (12–16 g/dL), she was urgently intubated and transferred to theatre for delivery of the retained placenta.

**Figure 1:**
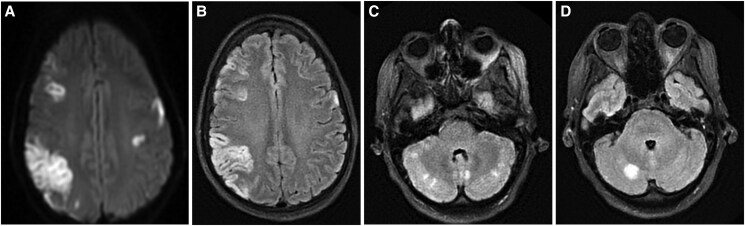
Brain magnetic resonance imaging. (*A*) Diffusion-weighted imaging and (*B*) T2 FLAIR (fluid attenuated inversion recovery) sequences showing a large area of cortical infarction in the right parietal lobe and additional infarcts in the frontal lobes. (*C*) and (*D*) showing acute cerebellar infarcts.

She was subsequently admitted to the intensive care unit for multi-organ support following the development of disseminated intravascular coagulation characterized by thrombocytopaenia [59 × 10^9^/L (150–450 × 10^9^/L)], decreased fibrinogen levels of 1.3 g/L (1.7–4.1 g/L), increased prothrombin time of 17.2 s (9.8–11.3 s), and activated partial thromboplastin time of 117 s (21–29 s) with a significantly raised d-dimer of 35 mg/L (normal < 0.5 mg/L). Two days later, upon extubation, she exhibited difficulties in following commands and was diagnosed with speech apraxia by the stroke team. Brain magnetic resonance imaging (MRI) revealed acute extensive ischaemia involving both cerebral hemispheres and the cerebellum. There was a large area of cortical infarction in the right parietal lobe and additional infarcts in the frontal lobes, caudate nuclei, and left thalamus. The pattern of cortical involvement with subcortical sparring raised the possibility of a reversible vasoconstriction syndrome (*Figure 1*).

**Figure 2:**
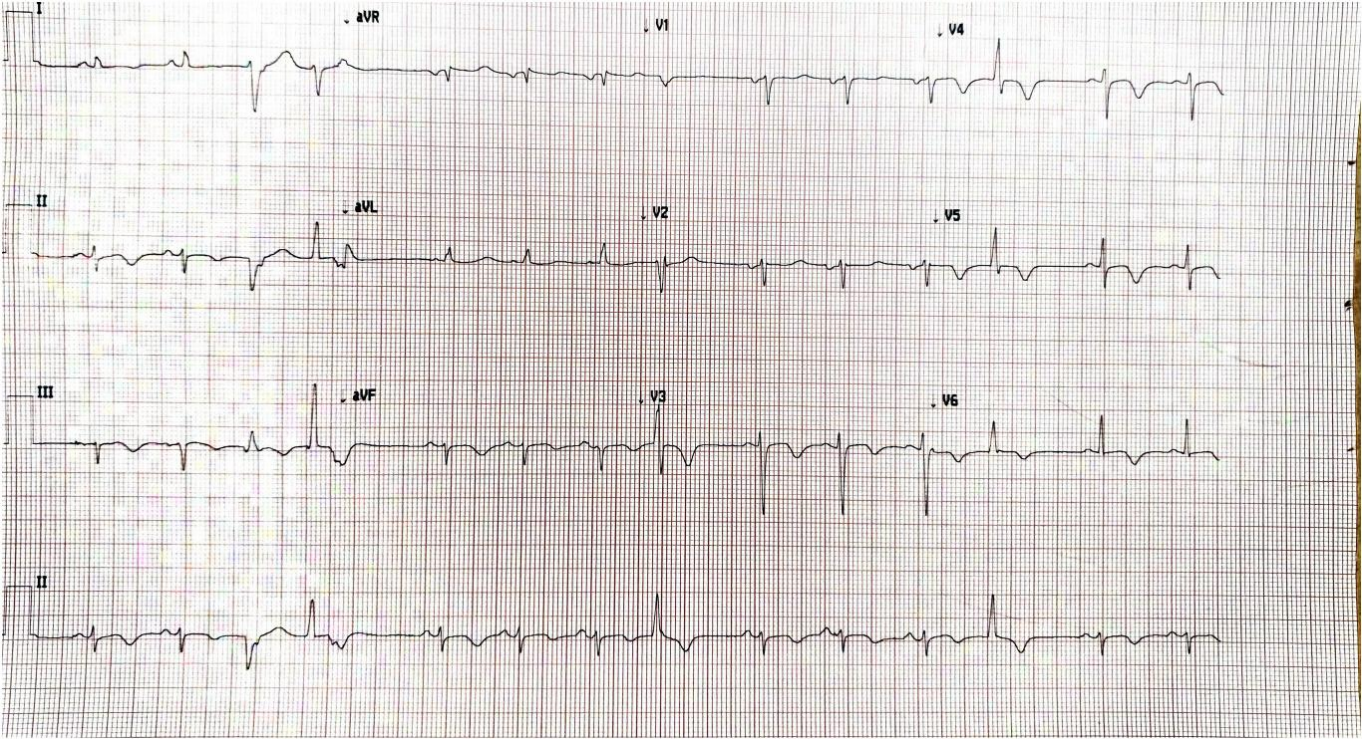
Electrocardiogram showing diffuse T-wave inversions.

**Figure 3:**
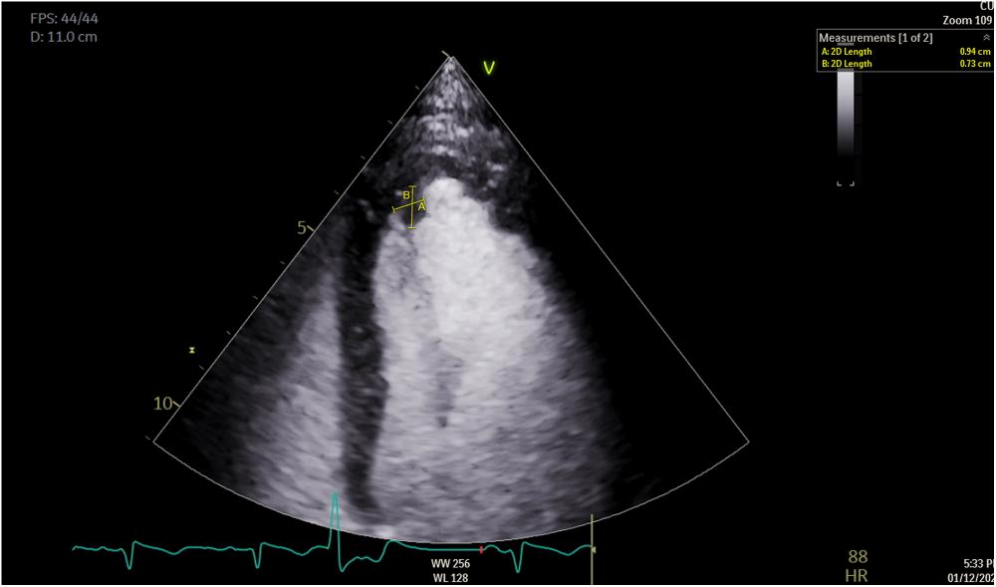
Focused contrast-echocardiography revealing small residual apical thrombus.

**Figure 4:**
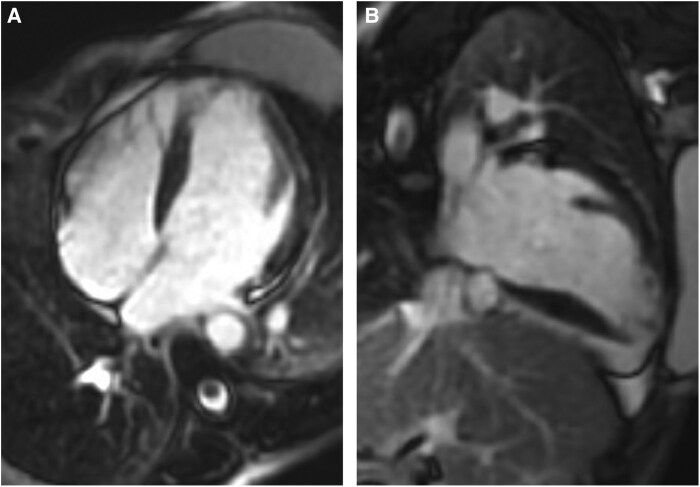
Cardiovascular MRI. (*A*) and (*B*) showing late-gadolinium enhancement (LGE) in the inferior and inferoseptal segments of the akinetic LV apex, suggestive of myocardial infarction.

Her electrocardiogram became noticeably abnormal with diffuse T-wave inversions (*Figure 2*). A high-sensitivity troponin-I was recorded at 37 000 ng/L (normal < 16 ng/L). Transthoracic echocardiography showed severe left ventricular (LV) systolic impairment, apical akinesia, and visible mobile echogenicity suggestive of thrombi (see Supplementary material online, *Video S1*). Therapeutic low-molecular weight heparin was initiated alongside heart failure (HF) therapy. One week later, she suffered from a second stroke due an acute middle cerebral artery (MCA) occlusion, resulting in aphasia and right-sided hemiparesis which required an emergency thrombectomy. Focused contrast-echocardiography revealed only small residual apical thrombus (*Figure 3*). Cardiovascular MRI showed late-gadolinium enhancement (LGE) in the inferior and inferoseptal segments of the akinetic LV apex, consistent with infarction. LV ejection fraction was 35%–40% (*Figure 4*).

**Figure 5:**
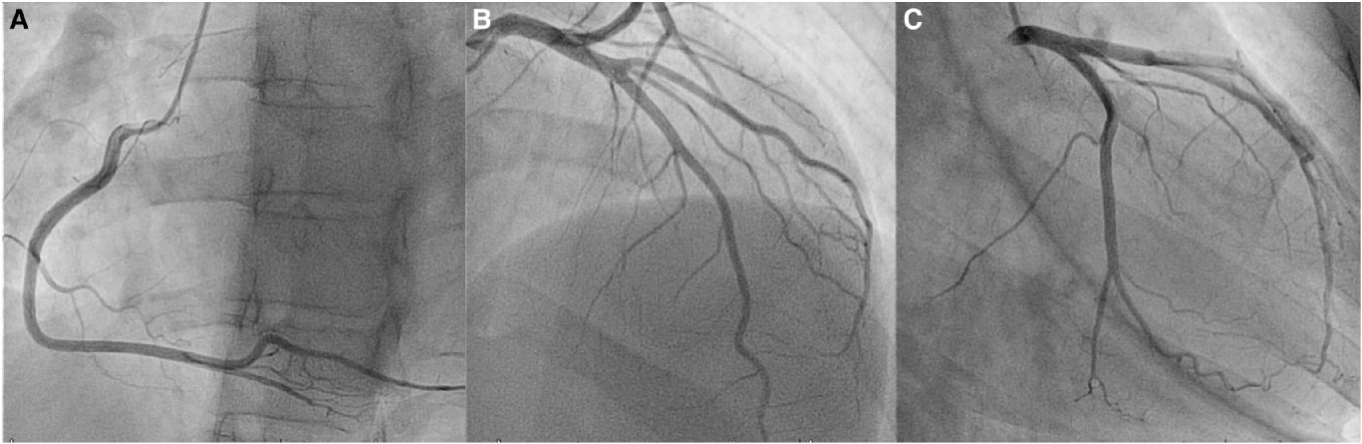
Invasive coronary angiography. (*A–C*) confirmed the presence of normal coronary arteries.

Cardiac catheterization excluded spontaneous coronary artery dissection (SCAD) and confirmed the presence of normal coronary arteries (*Figure 5* and Supplementary material). Despite the established safety of provocative testing, the patient expressed significant concerns about the risks and potential complications of inducing coronary vasospasm. Therefore, we decided to perform only a diagnostic coronary angiogram on that day, prioritizing patient’s wishes while still achieving diagnostic objectives.

After three weeks of hospitalization with extensive multi-disciplinary input, her speech and motor abilities significantly improved. Her thrombophilia profile was unremarkable, including normal levels of antithrombin, protein C, and protein S along with a negative antiphospholipid screen. Additionally, there was no evidence of Factor V Leiden or prothrombin G20210A gene mutations. She was discharged on isosorbide mononitrate, oral anticoagulation, and maximum-tolerated HF therapy including ramipril 1.25 mg, spironolactone 12.5 mg, dapagliflozin 10 mg, and bisoprolol 2.5 mg once daily. A clinic review two months later confirmed full recovery of motor function. Follow-up echocardiography revealed a significant improvement in LV ejection fraction (45%–50%) with complete resolution of the LV thrombus.

## Discussion

The differential diagnosis for acute LV dysfunction and subsequent strokes in the setting of life-threatening PPH included peripartum and Takotsubo cardiomyopathy (TCM). However, the elevation of troponin-I was significantly higher than typically seen in TCM and the presence of LV thrombi is uncommon. Coronary artery embolization and epicardial plaque rupture were also considered. The presence of focal LGE on cardiovascular MRI in a discrete territory coupled with angiographically normal coronaries and evidence of cerebral vasoconstriction pointed towards vasospasm as the most likely unifying explanation. The second MCA stroke was likely cardio-embolic due to the formation of multiple LV thrombi driven by Virchow’s triad of hypercoagulability, endothelial injury, and LV apical haemostasis.^3^

Following extensive multi-disciplinary discussions, the consensus of the most likely diagnosis was severe ergometrine-induced vasospasm, potentially causing simultaneous strokes and myocardial infarction. Haemorrhagic shock may have exacerbated the severity of AMI by worsening myocardial oxygen demand-supply mismatch during vasospasm.^4^

Ergometrine is part of the current guideline-directed management of PPH, acting directly on uterine smooth muscles to induce uterotonic contractions in cases of uterine atony.^5^ Despite the widespread use of ergometrine, only a few cases of vasospasm leading to AMI have been documented.^2^ While the precise mechanism remains unclear, several risk factors have been postulated including migraines, alcohol misuse, smoking, and pregnancy over the age of 30.^6,7^

While acute coronary syndromes (ACSs) during pregnancy are relatively uncommon (1.7–6.2 per 100 000 deliveries), pregnancy-associated MI (PAMI) is responsible for more than 5% of maternal cardiac mortality.^8–10^ Distinct from the general population, the majority of PAMI cases are driven by non-atherosclerotic mechanisms such as SCAD (43%) and coronary thrombosis (17%) and nearly 18% exhibit angiographically normal coronary arteries.^8,11^ Coronary vasospasm accounts for 2%–5% of PAMI and is likely triggered by elevated renin and angiotensin levels.^8,12^

Suspected coronary vasospasm in pregnancy should be treated promptly with nitroglycerine.^8,13^ According to the European Society of Cardiology (ESC) guidelines, low-risk PAMI patients with non-ST-elevation-ACS should be managed using an ischaemia-guided, non-invasive strategy.^14^ However, risk assessment tools such as the Global Registry of Acute Coronary Events (GRACE) score have not been validated for PAMI.^9,14^ Computed tomographic coronary angiography should be considered in low-risk patients, although limitations include the risk of foetal radiation, high-dose beta-blockers, and the possibility of inconclusive findings due to limited resolution or motion artefact.^15^ Nonetheless, understanding the aetiology of PAMI is important for guiding long-term care, even if revascularization is not performed. Consequently, invasive coronary angiography should be considered unless the projected risks outweigh the anticipated benefits in low-risk patients.

The ESC recommends extensive preconception counselling and a delay in conception for 12 months after PAMI.^14^ The LV function and ACS aetiology are the primary factors influencing safety. Subsequent pregnancies are contraindicated in individuals with severe LV dysfunction and those with a history of SCAD.^14^

## Conclusion

This case emphasizes the importance to recognize ACS as a significant cause of maternal morbidity and mortality. The early involvement of a multi-disciplinary cardio-obstetrics team and the use of multi-modal cardiac imaging to establish the diagnosis of coronary vasospasm is crucial. While ergometrine is widely used and generally safe in managing obstetric emergencies, a small subset of patients without typical cardiac risk factors may encounter severe cardiovascular complications and therefore should involve a degree of caution and close observation to reduce delay in diagnosis and management.

## Supplementary Material

ytaf087_Supplementary_Data

## Data Availability

The data underlying this article are available in the article and in its online Supplementary material.

## Lead author biography

Dr Avinash Radhakrishna is a Cardiology Specialist Registrar and a lecturer at University College Cork, Ireland. He graduated with honours from the National University of Ireland, Galway (NUIG) in 2017 and subsequently completed his Membership of the Royal College of Physicians of Ireland (MRCPI) in 2019. Dr Radhakrishna also earned a Master’s in Interventional Cardiology from NUIG in 2021, graduating with first-class honours. With a strong commitment to both clinical excellence and academic mentorship, Dr Radhakrishna is dedicated to training as an interventional cardiologist in Ireland while actively contributing to the development of future healthcare professionals through scholarly activity.

## Notes

**Consent:** The authors confirm that written consent for submission and publication of this case report including images and associated text has been obtained from the patient in line with the Committee on Publication Ethics (COPE) guidelines.

**Funding:** None.

## References

[1] Heesen M, Carvalho B, Carvalho JCA, Duvekot JJ, Dyer RA, Lucas DN, et al International consensus statement on the use of uterotonic agents during caesarean section. Anaesthesia 2019;74:1305–1319. 31347151 10.1111/anae.14757

[2] Spencer SPE, Lowe SA. Ergometrine for postpartum hemorrhage and associated myocardial ischemia: two case reports and a review of the literature. Clin Case Rep 2019;7:2433–2442. 31893076 10.1002/ccr3.2516 PMC6935658

[3] Delewi R, Zijlstra F, Piek JJ. Left ventricular thrombus formation after acute myocardial infarction. Heart 2012;98:1743–1749. 23151669 10.1136/heartjnl-2012-301962 PMC3505867

[4] Byrne RA, Rossello X, Coughlan JJ, Barbato E, Berry C, Chieffo A, et al 2023 ESC Guidelines for the management of acute coronary syndromes. Eur Heart J 2023;44:3720–3826. 37622654 10.1093/eurheartj/ehad191

[5] Mavrides E, Allard S, Chandraharan E, Collins P, Green L, Hunt BJ, et al Prevention and management of postpartum haemorrhage: green-top guideline no. 52. BJOG 2017;124:e106–e149. 27981719 10.1111/1471-0528.14178

[6] Taylor GJ, Cohen B. Ergonovine-induced coronary artery spasm and myocardial infarction after normal delivery. Obstet Gynecol 1985;66:821–822. 3877894

[7] Hokimoto S, Kaikita K, Yasuda S, Tsujita K, Ishihara M, Matoba T, et al JCS/CVIT/JCC 2023 guideline focused update on diagnosis and treatment of vasospastic angina (coronary spastic angina) and coronary microvascular dysfunction. Circ J 2023;87:879–936. 36908169 10.1253/circj.CJ-22-0779

[8] Elkayam U, Jalnapurkar S, Barakkat MN, Khatri N, Kealey AJ, Mehra A, et al Pregnancy-associated acute myocardial infarction: a review of contemporary experience in 150 cases between 2006 and 2011. Circulation 2014;129:1695–1702. 24753549 10.1161/CIRCULATIONAHA.113.002054

[9] Tweet MS, Lewey J, Smilowitz NR, Rose CH, Best PJM. Pregnancy-associated myocardial infarction: prevalence, causes, and interventional management. Circ Cardiovasc Interv 2020;13:Circinterventions120008687 11. 10.1161/CIRCINTERVENTIONS.120.008687 PMC7854968 32862672

[10] Ladner HE, Danielsen B, Gilbert WM. Acute myocardial infarction in pregnancy and the puerperium: a population-based study. Obstet Gynecol 2005;105:480–484. 15738011 10.1097/01.AOG.0000151998.50852.31

[11] Tweet MS, Hayes SN, Codsi E, Gulati R, Rose CH, Best PJM. Spontaneous coronary artery dissection associated with pregnancy. J Am Coll Cardiol 2017;70:426–435. 28728686 10.1016/j.jacc.2017.05.055

[12] Gant NF, Daley GL, Chand S, Whalley PJ, MacDonald PC. A study of angiotensin II pressor response throughout primigravid pregnancy. J Clin Invest 1973;52:2682–2689. 4355997 10.1172/JCI107462 PMC302534

[13] Knuuti J, Wijns W, Saraste A, Capodanno D, Barbato E, Funck-Brentano C, et al 2019 ESC Guidelines for the diagnosis and management of chronic coronary syndromes. Eur Heart J 2020;41:407–477. 31504439 10.1093/eurheartj/ehz425

[14] Regitz-Zagrosek V, Roos-Hesselink JW, Bauersachs J, Blomström-Lundqvist C, Cífková R, De Bonis M, et al 2018 ESC Guidelines for the management of cardiovascular diseases during pregnancy. Eur Heart J 2018;39:3165–3241. 30165544 10.1093/eurheartj/ehy340

[15] Tweet MS, Gulati R, Williamson EE, Vrtiska TJ, Hayes SN. Multimodality imaging for spontaneous coronary artery dissection in women. JACC Cardiovasc Imaging 2016;9:436–450. 27056163 10.1016/j.jcmg.2016.01.009

