# Supplementary material for: Joining forces: a complex cardio-obstetrics case report of severe ergometrine-induced vasospasm
Source: Eur Heart J Case Rep. 2025 Feb 18;9(3):ytaf087. doi: 10.1093/ehjcr/ytaf087 (PMC11879452; doi:10.1093/ehjcr/ytaf087)
Supplement: ytaf087_Supplementary_Data [file ytaf087_supplementary_data.zip › Legend for Supplementary Materials.docx]

**Supplementary Material: Video Legends**

**Video S1:**Transthoracic echocardiography showing severe left ventricular systolic impairment, apical akinesia and visible mobile echogenicity suggestive of thrombi

**Video S2:** Coronary angiography showing angiographically normal coronary arteries

**Video S2.1:** Left anterior oblique (LAO) view of the right coronary artery

**Video S2.2:** Right anterior oblique (RAO) view of the left coronary artery

**Video S2.3:** Right anterior oblique (RAO) caudal view of the left coronary artery

**Video S2.4:** Posterior-anterior (PA) cranial view of the left coronary artery

**Video S2.5:** Left anterior oblique (LAO) caudal view of the left coronary artery
